# Risk matters for atherosclerotic cardiovascular disease: But vascular resilience may decide

**DOI:** 10.1016/j.ajpc.2025.101348

**Published:** 2025-11-18

**Authors:** Michael D. Shapiro, Khurram Nasir

Preventive cardiology is built on a powerful and productive truth. Cumulative exposure to risk factors drives atherosclerosis. The logic is straightforward. Elevated levels of circulating atherogenic lipoproteins, hypertension, smoking, metabolic injury, and inflammation damage the arterial wall over time. Reduce exposure early and aggressively and events fall. And while this framework has fueled major advances in risk prediction and therapies that prevent heart attacks and strokes and it saved millions of lives, we must now ask a fundamental question. Is it the whole story?

Clinical practice and emerging research tells us it is not. Risk exposure does not affect all arteries equally. Every clinician has cared for patients who defy expectation. One patient carries every major risk factor yet shows minimal or no plaque. Another develops diffuse atherosclerosis despite only modest traditional risk. These individuals are not statistical anomalies. They are biological evidence that risk alone does not determine outcome. They are a reflection of a missing dimension in our model: the vascular response to risk. In order words, vascular resilience.

Two people may share identical LDL cholesterol levels yet follow entirely different vascular trajectories. One develops progressive, inflamed plaque and clinical events. The other maintains stable, durable arterial health for decades. One artery succumbs easily to injury, another resists. Our field has been organized around risk but not around response to risk. We have built a science of vulnerability without building a science of protection. We have pursued the causes of arterial injury without pursuing the causes of arterial defense. Until we study both, prevention will remain one-dimensional.

## Vascular heterogeneity is real

The assumption that similar exposure yields similar disease is disproven daily in clinical practice. A 59-year-old man with an untreated LDL-cholesterol above 200 mg/dL for decades has a coronary calcium score of zero. A 44-year-old woman with normal blood pressure, normal BMI, no diabetes, and an LDL of 115 mg/dL has multivessel coronary artery disease. A 70-year-old former smoker with a calcium score above 1000 remains event-free for more than a decade. These findings challenge the one-dimensional, reductionist framework that still dominates risk prediction. They ask us to move toward a systems biology approach, where genetics, environment, inflammation, vascular structure, and resilience are all part of the equation.

Coronary artery calcium scanning first exposed this heterogeneity by showing the wide distribution of plaque burden at every level of risk. Coronary imaging soon revealed heterogeneity in plaque behavior—some lesions rupture early, others calcify and quiet. Inflammatory studies showed heterogeneity in vascular immune response. These are not curiosities; they are clinical consequences of unmeasured biology. Yet our prevention framework still treats risk exposure as if it were biology itself. It is not.

## What is vascular resilience & why?

Vascular resilience is the intrinsic ability of the arterial system to resist, adapt to, and recover from atherogenic stress over time. It can operate through three primary mechanisms:

1. Resistance to plaque initiation
  Some individuals simply do not form meaningful plaque despite prolonged exposure. Endothelial barrier remains intact, lipoprotein retention is low, shear stress regulation is preserved, and protective inflammatory mechanisms remain intact .
2. Control of inflammatory tone
  Resilience does not imply the absence of inflammation. It implies the ability to resolve it. After exposure to metabolic or immune triggers, resilient arteries restore equilibrium instead of remaining in smoldering inflammatory activation. Macrophage behavior shifts toward repair and clearance rather than necrotic expansion. Vascular injury does not escalate, favoring repair over necrosis.
3. Structural stability of plaque
  Resilience also protects when disease exists. Stable plaques with thick caps and limited lipid cores remain biologically quiet and event resistant

Resilience is not a myth. Its measurable biology, but yet not fully modeled. The implications of not considering it can be profound: It explains why not all risk is equal. Two patients with the same LDL-C or Lp(a) may not have the same biological vulnerability. Resilience supports precision prevention. Treatment intensity should reflect both exposure and response. It makes prevention proactive rather than reactive. Risk reduction prevents events. Resilience preservation extends vascular health.

## Medicine advances when we study those who resist

Cardiology has always advanced by studying extreme phenotypes. Familial hypercholesterolemia revealed LDL receptor biology. PCSK9 loss-of-function variants revealed a therapeutic pathway. CETP deficiency reshaped understanding of HDL. Now another extreme phenotype waits to be studied: people who do not develop atherosclerosis despite obvious exposure. They are not statistical noise. They are biological models of success. Yet our research infrastructure ignores them. We study only those who develop disease. We must also study those who avoid it.

Imagine if prevention science:

• Identified individuals who are plaque-resistant
• Characterized event-resistant biology
• Tracked resilience trajectories over decades
• Built resilience markers the way CAC became a burden marker

## A two-dimensional model for prevention

Risk estimation is necessary but insufficient. Prevention must evolve from a one-dimensional exposure model to a two-dimensional vascular model:

Risk Exposure + Vascular Response → True Disease *Probability*

This model allows us to a) identify patients who are biologically protected despite risk factor exposure, b) personalize treatment intensity based not just on risk, but on disease expression, c) deliver more efficient and targeted care, and d) optimize clinical trial design by avoiding overtreatment of resilient phenotypes

If this field chooses to evolve, it will mean three changes in mindset. First, we must treat resilience as biology, not mystery. We cannot keep explaining disease resistance as luck. For this we need to pivot our focus on measuring the arterial wall directly beyond bloodstream metrics of exposure Third, we must think beyond risk snapshots. Atherosclerosis is dynamic. Prevention must track direction - who is progressing, who is stable, who is reversing disease. While we have spent decades focused on risk factor quantity, the future of prevention will require greater attention to vascular quality.

## Where ASPC & AJPC stands

At this critical inflection point, both the American Society for Preventive Cardiology (ASPC) and the *American Journal of Preventive Cardiology (AJPC)* are aligned in our vision to elevate the science, clarity, and impact of prevention. As the President and EIC, we are committed to expanding prevention beyond its traditional focus on risk management. This does not mean we abandon risk; it means we complete the picture. The next generation of preventive cardiologists must be trained not only to calculate risk but to interpret vascular behavior, plaque biology, and long-term disease trajectories. Prevention will advance when we learn to protect arteries, not just correct numbers.

ASPC, will continue to invest in education, mentorship, and policy that empowers our members to integrate these ideas into practice and advocate for a broader, biology-driven definition of prevention. In parallel, AJPC will actively seek and publish work that addresses these gaps, from studies on imaging-based resilience markers, to longitudinal tracking of vascular adaptation, to mechanistic and translational research on protection to push this conversation forward

The next major step in cardiovascular disease prevention will not be found in a guideline revision or another risk equation. It will be found in biology, in understanding why some arteries resist disease. We have studied vulnerability long enough. Now we must also study protection. Somewhere inside the biology of those who stay healthy, despite years of exposure, lies the future of prevention. They are not anomalies. They are not lucky. They are resilient.

We invite the entire field - clinicians, scientists, educators, funders - to join us in building this future. ASPC and AJPC are committed to serving as platforms where this vision can take root and grow**.**

Because in the end: Risk matters. Resilience decides. It is time we study both.

## Author declaration

I wish to draw the attention of the Editor to the following facts which may be considered as potential conflicts of interest and to significant financial contributions to this work, as follows:

Michael D. Shapiro has received grant/research support (through his institution) from Amgen, Arrowhead, Boehringer Ingelheim, Esperion, Novartis, Ionis, Merck, New Amsterdam, Lilly, and Cleerly. He has participated in Scientific Advisory Boards with Amgen, Arrowhead, Ionis, Novartis, New Amsterdam, and Merck. He has served as a consultant for Ionis, Novartis, Regeneron, Aidoc, Shanghai Pharma Biotherapeutics, Kaneka, Novo Nordisk, Arrowhead, and Tourmaline.

Khurram Nasir has served as a consultant and scientific advisor for Amgen, New Amsterdam, Merck, Regeneron, and Corsera Health.

I confirm that the manuscript has been read and approved by both authors, the sole author and that there are no other persons who satisfied the criteria for authorship but are not listed. I further confirm that the order of authors listed in the manuscript has been approved by me, the sole authors. I confirm that I have given due consideration to the protection of intellectual property associated with this work and that there are no impediments to publication, including the timing of publication, with respect to intellectual property. In so doing, I confirm that I have followed the regulations of our institutions concerning intellectual property.

I understand that the Corresponding Author is the sole contact for the Editorial process (including Editorial Manager and direct communications with the office). He/she is responsible for communicating with the other authors about progress, submissions of revisions and final approval of proofs. I confirm that I have provided a current, correct email address which is accessible by the Corresponding Author.

## CRediT authorship contribution statement

**Michael D. Shapiro:** Conceptualization, Writing – original draft, Writing – review & editing. **Khurram Nasir:** Writing – review & editing.

## Declaration of competing interest

The authors declare the following financial interests/personal relationships which may be considered as potential competing interests: Michael D. Shapiro reports a relationship with Amgen, Arrowhead, Ionis, Novartis, New Amsterdam, Merck, Regeneron, Aidoc, Novo Nordisk, and Tourmaline that includes: consulting or advisory. Michael D. Shapiro reports a relationship with Amgen, Arrowhead, Boehringer Ingelheim, Esperion, Novartis, Ionis, Merck, New Amsterdam, Lilly, and Cleerly that includes: funding grants. If there are other authors, they declare that they have no known competing financial interests or personal relationships that could have appeared to influence the work reported in this paper. Khurram Nasir reports a relationship with Amgen, New Amsterdam, Merck, Regeneron, and Corsera Health that includes: consulting or advisory.

